# How do global policy frameworks address the ethics of pain management? A qualitative content analysis of WHO and WMA documents

**DOI:** 10.1136/bmjopen-2025-111913

**Published:** 2026-05-07

**Authors:** Sevim Coşkun, Nüket Örnek Büken

**Affiliations:** 1 History of Medicine and Medical Ethics, Hacettepe University Faculty of Medicine, Ankara, Turkey

**Keywords:** PALLIATIVE CARE, PAIN MANAGEMENT, MEDICAL ETHICS, Health policy, Health Equity, Health Services Accessibility

## Abstract

**Abstract:**

**Objectives:**

To examine how the WHO and the World Medical Association (WMA) frame bioethical principles and address implementation barriers in their pain management policies, providing insights for global health policy and ethical analysis.

**Design:**

Qualitative content analysis of international policy documents using the Standards for Reporting Qualitative Research to ensure methodological transparency and analytical rigour.

**Data sources:**

Analysis of publicly available policy documents produced by the WHO and WMA between January 2000 and December 2024.

**Eligibility criteria:**

Documents addressing pain management with ethical content, current and not superseded (n=18 from 314 screened).

**Data extraction and synthesis:**

18 policy documents were retrieved through relevance screening and analysed with reference to ethical values and systemic constraints using MAXQDA Analytics Pro 2022. Thematic coding identified ethical principles, structural barriers and strategic policy directions shaping global pain management frameworks.

**Results:**

Nine ethical principles underpin global pain management policies, including human rights-based access, professional duty to relieve suffering and equitable care. Seven major barriers, such as regulatory restrictions, educational deficiencies and systemic inequities, hinder implementation. Five policy directions were identified to bridge principles and practice.

**Conclusions:**

WHO and WMA frameworks articulate a shared normative commitment to equitable, safe and person-centred pain management but differ in emphasis between public health and clinical ethics perspectives. Addressing identified structural barriers, integrating biopsychosocial approaches, and promoting culturally sensitive ethical guidance are critical for improving global pain management policies. While international guidelines provide the ethical foundations, achieving equitable global pain care requires coordinated transformation across regulatory, educational and health system domains. The persistent gap between ethical commitments and real-world implementation underscores the urgent need for binding accountability mechanisms, stronger international coordination and systematic approaches to address structural determinants of inequity.

**STRENGTHS AND LIMITATIONS OF THIS STUDY**

- Applies a rigorous qualitative content analysis of 18 WHO and World Medical Association (WMA) policy documents, providing a systematic mapping of ethical frameworks in global pain governance.
- Adheres to the Standards for Reporting Qualitative Research, ensuring methodological transparency and analytical coherence.
- Combines inductive and deductive coding to integrate empirical findings with established bioethical and human rights principles.
- Offers an original synthesis linking ethical theory and policy practice in pain management.
- Limited to WHO and WMA documents and does not assess national or clinical implementation outcomes.

## Introduction

 Effective pain management is a key determinant of quality of life and is widely recognised as a fundamental human right by international organisations, such as the International Association for the Study of Pain (IASP), the WHO and the World Medical Association (WMA).^1^,^5^ Despite this recognition, both chronic and acute pain affect people globally, and access to timely, person-centred and cost-effective pain management remains unattainable for most, particularly in rural and underserved regions.^6^ The burden spans diverse pain types (including acute pain from injuries or procedures, cancer-related pain and persistent chronic pain conditions), yet remains especially high in low- and middle-income countries (LMICs), where multidisciplinary pain management services are often lacking due to limited resources, low awareness and insufficiently trained healthcare professionals.^7 8^

The IASP’s contemporary definition recognises pain as ‘an unpleasant sensory and emotional experience associated with, or resembling that associated with, actual or potential tissue damage’, explicitly acknowledging that pain is influenced by biological, psychological and social factors.^9^ This biopsychosocial conceptualisation underscores that effective pain management requires comprehensive, person-centred approaches addressing multiple dimensions of suffering simultaneously, a framework that should inform global policy development.

These persistent implementation gaps reflect systemic barriers, including restrictive legal frameworks that often exceed international drug convention requirements; limited opportunities for professional training in pain assessment and management, including opioid pharmacology; socioeconomic disadvantages and enduring stigma surrounding the use of controlled substances.^2^,^510^

This study aims to address this implementation challenge by investigating how ethical principles are articulated within guidelines related to pain management produced by the WHO and WMA and critically examining how these normative frameworks address, or fail to address, the structural and regulatory barriers that drive the implementation gap.

## Methods

This study followed the Standards for Reporting Qualitative Research, as recommended by the Enhancing the QUAlity and Transparency Of health Research (EQUATOR) Network, to ensure complete and transparent reporting of qualitative research practices.^21 22^ We conducted qualitative content analysis (QCA) of 18 WHO and WMA policy documents between January 2000 and December 2024, selected through relevance screening based on predefined inclusion criteria.

### Study design and rationale

This study employed QCA, a systematic and adaptable method commonly used to examine textual material in health-related research.^21 22^ The analysis employed a two-phase approach: initial inductive coding to identify emerging themes directly from document content, followed by deductive refinement incorporating established concepts from medical ethics and human rights literature to enhance conceptual clarity.

### Document retrieval and sampling strategy

Two researchers (SC and NOB) retrieved documents from WHO and WMA official websites. The timeframe 2000–2024 was selected to capture the current pain policy frameworks. For WMA, the complete 2025 *Handbook of WMA Policies* was reviewed to confirm the current status of policies adopted prior to 2025, encompassing 205 documents (37 declarations, 111 statements and 57 resolutions).^23^

WHO documents in English were identified through keyword searches using terms including “pain management,” “palliative care” and “opioid” supplemented by snowball sampling whereby references and hyperlinks within initially identified documents were systematically followed. This process yielded 109 WHO documents deemed relevant to the study scope (online supplemental file 1).

Palliative care documents feature prominently in our corpus because WHO’s pain management guidance has historically developed primarily within palliative and cancer care contexts, where pain relief represents a core therapeutic objective. While palliative care traditionally focuses on life-limiting illness rather than chronic non-cancer pain, many ethical principles (human rights to pain relief, equitable access and balanced regulation) and implementation barriers (regulatory restrictions and educational gaps) apply across diverse pain management contexts. Thus, palliative care-focused documents provide valuable insights into broader pain management ethics, even as we acknowledge that chronic non-cancer pain may raise distinct ethical considerations requiring additional policy attention.

### Inclusion criteria and document selection

From 314 screened documents, inclusion criteria required: (1) explicit reference to pain management or related symptom control, (2) engagement with ethical principles, values or dilemmas and (3) continued policy relevance (not formally withdrawn or superseded). Documents focused solely on clinical content without normative framing were excluded. This process yielded 18 highly relevant policy documents representing authoritative international guidance (table 1).

**Table 1:** Included policy documents (n=18), listed in reverse chronological order by organisation

No.	Organisation	Title of guideline or policy document	Publication year
1	WHO	Left Behind in Pain: Extent and Causes of Global Variations in Access to Morphine for Medical Use and Actions to Improve Safe Access	2023
2	WHO	WHO Guideline for Non-Surgical Management of Chronic Primary Low Back Pain in Adults in Primary and Community Care Settings	2023
3	WHO	Consolidated Guidelines on HIV Prevention, Testing, Treatment, Service Delivery and Monitoring: Recommendations for a Public Health Approach	2021
4	WHO	Guidelines on the Management of Chronic Pain in Children	2020
5	WHO	Written Statements Public Hearing on Guideline Scoping Document: WHO Guideline on Ensuring Balanced National Policies for Access and Safe Use of Controlled Medicines	2020
6	WHO	Integrating Palliative Care and Symptom Relief into Paediatrics	2018
7	WHO	Integrating Palliative Care and Symptom Relief into Primary Health Care	2018
8	WHO	Integrating Palliative Care and Symptom Relief into the Response to Humanitarian Emergencies and Crises: A WHO Guide	2018
9	WHO	WHO Guidelines for the Pharmacological and Radiotherapeutic Management of Cancer Pain in Adults and Adolescents	2018
10	WHO	Planning and Implementing Palliative Care Services: A Guide for Programme Managers	2016
11	WHO	Comprehensive Cervical Cancer Control: A Guide to Essential Practice – Second Edition	2014
12	WHO	Strengthening of Palliative Care as a Component of Integrated Treatment Throughout the Life Course	2014
13	WHO	Guidelines for the Psychosocially Assisted Pharmacological Treatment of Opioid Dependence	2009
14	United Nations, WHO, International Narcotics Control Board	Guide on Estimating Requirements for Substances under International Control	2012
15	WMA	WMA Declaration of Venice on End of Life Medical Care	2022
16	WMA	WMA Declaration of Ottawa on Child Health	2020
17	WMA	WMA Resolution on the Access to Adequate Pain Treatment	2020
18	WMA	WMA Statement on the Responsibilities of Physicians in Preventing and Treating Drug Abuse	2016

WMA, World Medical Association.

### Data processing and analysis

All selected documents were imported into MAXQDA Analytics Pro 2022, chosen for its robust support of QCA, including coding, memo creation and thematic visualisation.^22^ SC conducted initial inductive coding through close reading of all documents, developing codes around recurring themes, including main bioethical principles (beneficence, non-maleficence, autonomy and justice),^24^ human rights concepts and implementation barriers (stigma, access restrictions and resource limitations).

The coding framework was subsequently reviewed and refined by NOB through independent parallel coding of a subset of documents, with systematic comparison and discussion of coding decisions. In the deductive phase, codes were reviewed to enhance theoretical grounding. Both researchers collaboratively developed the final codebook through iterative discussion, ensuring thematic coherence and analytical rigour (online supplemental file 2).

### Conceptual framework for ethical principles

Following Beauchamp and Childress’s principlist approach,^24^ we distinguished between foundational ethical principles (core moral commitments such as beneficence, non-maleficence, autonomy and justice) and derivative ethical obligations (specific duties that emerge from these foundational principles in the context of pain management policy). Our analysis identifies both types, recognising that policy documents often articulate principles at varying levels of abstraction, from broad normative commitments to specific implementation-oriented guidance.

### Researcher characteristics

Both researchers are medical doctors with academic training in medical ethics (SC: PhD candidate; NOB: professor of medical ethics).

#### Patient and public involvement

Patients and/or the public were not involved in the design, conduct, reporting or dissemination plans of this research.

## Results

### Overview of documents analysed

18 documents were included in the final analysis: 13 from the WHO and five from the WMA. These encompassed technical guidelines, public health frameworks, ethical declarations and policy statements. All documents were current at the time of analysis and addressed pain management either directly or indirectly.

The QCA identified nine core ethical principles, seven major barriers and five strategic policy directions. These constitute the primary thematic categories shaping global discourse on pain care. Three cross-cutting themes emerged across all categories: equity as a foundational value, human rights frameworks as policy anchors and health system integration as an implementation imperative.

### Main ethical principles in pain management

The analysis revealed a coherent ethical framework underpinning the WHO and WMA guidance documents, identifying nine core ethical principles. Following our conceptual framework, these are categorised into foundational principles (eg, access as a human right and equitable access), which map directly to bioethical justice and rights theories, and derivative professional obligations (eg, interdisciplinary approaches and commitment to safety), which represent the practical application of beneficence and non-maleficence in clinical systems. Collectively, they reflect both clinical duties and broader commitments to human rights, professional responsibility and equitable healthcare.

**Figure 1:**
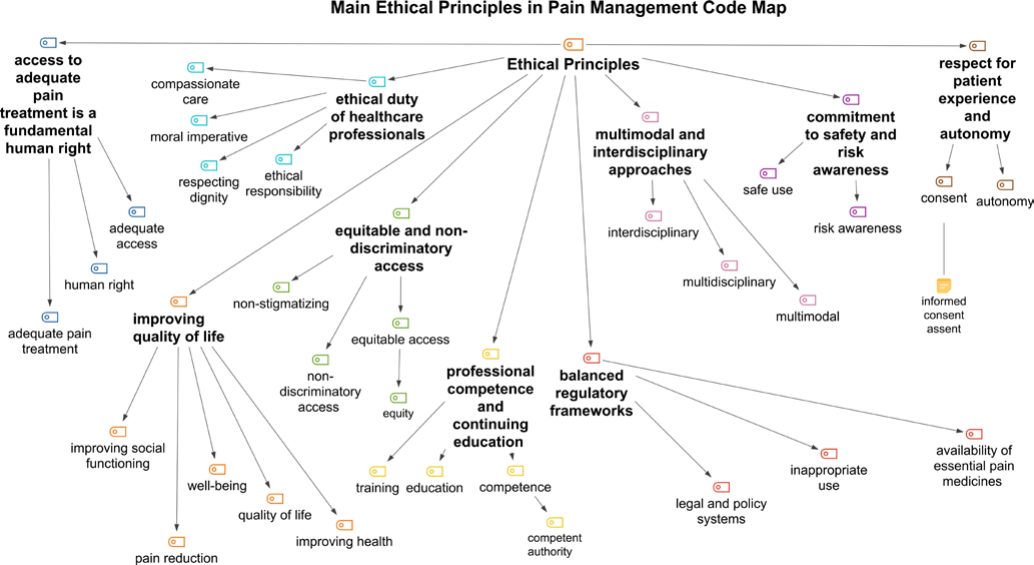
Hierarchical code map of ethical principles in pain management. This figure presents a hierarchical visualisation of the ethical principles identified through qualitative content analysis. The coding structure was developed using MAXQDA Analytics Pro 2022 and reflects the relationships between overarching ethical themes, sub-dimensions and analytical categories within the WHO and WMA documents. WMA, World Medical Association.

Figure 1 illustrates the hierarchical structure of ethical principles identified in our analysis. Notably, the framework’s architecture reflects both strengths and limitations of current policy discourse. The prominence of human rights language and access principles demonstrates normative evolution beyond purely clinical frameworks. However, the figure also reveals potential conceptual tensions: principles such as ‘balanced regulation’ and ‘safety commitment’ may conflict with ‘equitable access’ in practice, yet the documents provide limited guidance for resolving such conflicts. The relative emphasis on certain principles over others (for instance, extensive articulation of professional duties but limited attention to patient agency beyond formal autonomy) suggests areas where future policy development could strengthen normative frameworks.

#### Access to adequate pain treatment is a fundamental human right

Documents consistently frame access to adequate pain treatment as a fundamental human right. Both organisations stress that failing to ensure access, especially in palliative and end-of-life contexts, may constitute a violation of international legal and ethical standards. They also affirm that pain management is not merely a therapeutic option but a legal and ethical obligation grounded in human rights (table 2, Principle 1).

**Table 2:** Key quotations supporting ethical principles

Ethical principle	Organisation	Key quote	Reference
Principle 1: access to adequate pain treatment is a fundamental human right	WHO	'Recognising that palliative care (…) is fundamental to improving the quality of life, well-being, comfort and human dignity (…), and (…) access to (…) essential medicines (…), including opioid analgesics such as morphine, (…) contributes to the realisation of the right to the enjoyment of the highest attainable standard of health and well-being’.	WHO, Integrating Palliative Care and Symptom Relief into Paediatrics: A WHO Guide for Health-Care Planners, Implementers and Managers, 2018.
	WMA	'Patients in pain shall be given access to effective pain medication, including opioids. Depriving them of such right is a violation of their right to health and is medically unethical’.	WMA, Resolution on the Access to Adequate Pain Treatment, 2020.
Principle 2: pain relief as an ethical duty of healthcare professionals	WHO	'It is the ethical duty of health care professionals to alleviate pain and suffering, whether physical, psychosocial or spiritual, irrespective of whether the disease or condition can be cured’.	WHO, Integrating Palliative Care and Symptom Relief into Primary Health Care: A WHO Guide for Planners, Implementers and Managers, 2018.
	WMA	'Physicians should assist the dying patient in maintaining an optimal quality of life by controlling symptoms and addressing psychosocial and spiritual needs, to enable the patient to die with dignity and in comfort’.	WMA, Declaration of Venice on End of Life Medical Care, 2022.
Principle 3: promoting quality of life as a central objective of ethical pain care	WHO	‘The goal of pain management is to relieve pain to a level that allows for an acceptable quality of life’.	WHO, Guidelines for the Pharmacological and Radiotherapeutic Management of Cancer Pain in Adults and Adolescents, 2018.
	WMA	’In most cases, pain can be stopped or relieved with inexpensive and relatively simple treatment interventions, which can dramatically improve the quality of life for patients’.	WMA, Resolution on the Access to Adequate Pain Treatment, 2020.
Principle 4: equitable and non-discriminatory access to pain care	WHO	‘(…) access to morphine and other strong opioids is unequal and inadequate globally’.	WHO, Left Behind in Pain: Extent and Causes of Global Variations in Access to Morphine for Medical Use and Actions to Improve Safe Access, 2023.
	WMA	‘Patients in pain shall be given access to effective pain medication, including opioids. Depriving them of such right is a violation of their right to health and is medically unethical’.	WMA, Resolution on the Access to Adequate Pain Treatment, 2020.
Principle 5: professional competence and continuing education in pain care	United Nations (UN)–WHO–International Narcotics Control Board (INCB)	‘(…) insufficient education of health-care professionals as a barrier to opioid availability. These results call attention to the need for educational initiatives to address health-care professionals’ lack of knowledge about appropriate modern pain management with opioids, treatment of anxiety and insomnia with psychotropic substances, and treatment of other conditions with narcotic drugs and psychotropic substances’.	United Nations–WHO–International Narcotics Control Board, Guide on Estimating Requirements for Substances under International Control, 2012.
	WMA	‘Pain treatment and control education shall be provided to healthcare professionals (…). The curriculum shall be highly competence-based in design, enhancing the knowledge, the attitude, and the skills of healthcare professionals while treating pain’.	WMA, Resolution on the Access to Adequate Pain Treatment, 2020.
Principle 6: balancing access and control in pain medicine regulation	WHO	‘The failure to ensure access to controlled medicines for the relief of pain and suffering threatens fundamental rights to health and to protection against cruel, inhuman, and degrading treatment’.	WHO, Guidelines on the Management of Chronic Pain in Children, 2020.
	WMA	‘Governments must ensure that controlled drugs, including opioids, are made available and accessible to help relieve the suffering’.	WMA, Resolution on the Access to Adequate Pain Treatment, 2020.
Principle 7: interdisciplinary and multimodal approaches are ethically essential in pain care	WHO	‘Pain management thus requires a multimodal, interdisciplinary and integrated approach’.	WHO, Guidelines on the Management of Chronic Pain in Children, 2020.
	WMA	‘Sometimes, especially in severe chronic pain, psycho-emotional factors are even more significant than physiologic factors. Pain treatment in these cases may require a multi-faceted approach to care by multidisciplinary teams’.	WMA, Resolution on the Access to Adequate Pain Treatment, 2020.
Principle 8: commitment to safety and risk awareness in pain care	WHO	‘Policy-makers, programme managers and healthcare providers, as well as families and caregivers must attend to opioid stewardship to ensure the rational and cautious use of opioids’.	WHO, Guidelines on the Management of Chronic Pain in Children, 2020.
	WHO	‘Safety of patients, carers, health-care providers, communities and society must be assured’.	WHO, Guidelines for the Pharmacological and Radiotherapeutic Management of Cancer Pain in Adults and Adolescents, 2018.
	WMA	‘Physicians must take all necessary measures to ensure that they are fully informed of the effects of these drugs. This includes reviewing up-to-date research regarding dosage, potential effectiveness for the specific condition, potential side effects and pharmacological interactions, and prevalence of misuse’.	WMA, Statement on the Responsibilities of Physicians in Preventing and Treating Opiate and Psychotropic Drug Abuse, 2016.
Principle 9: respect for patient experience and autonomy	WHO	‘Treatment should respect and validate the autonomy of the individual, with patients being fully informed about the risks and benefits of treatment choices’.	WHO, Guidelines for the Psychosocially Assisted Pharmacological Treatment of Opioid Dependence, 2009.
	WMA	‘Ethically appropriate care at the end of life should routinely promote patient autonomy and shared decision-making, and be respectful of the values of the patient, his or her family or intimate associates, and surrogate(s)’.	WMA, Declaration of Venice on End of Life Medical Care, 2022.

WMA, World Medical Association.

#### Pain relief as an ethical duty of healthcare professionals

Healthcare professionals have an ethical responsibility to assess, prevent and relieve pain. This duty is grounded in both clinical competence and moral values, such as compassion, respect for dignity and the alleviation of suffering. These documents emphasise that pain relief is not optional but a professional and moral obligation requiring clinicians to act as both skilled practitioners and compassionate caregivers (table 2, Principle 2).

#### Promoting quality of life as a central objective of ethical pain care

Both organisations frame (table 2, Principle 3) pain management as a strategy to enhance quality of life, encompassing physical relief as well as psychological, social and spiritual well-being.

#### Equitable and non-discriminatory access to pain care

Documents present equitable access as a fundamental ethical obligation. Pain relief should be available regardless of socioeconomic status, geographic location, demographic characteristics or diagnosis. Such disparities are described not only as logistical failures but also as ethical and human rights concerns.

The WHO also notes that stigma and fear surrounding opioid use remain major obstacles, often resulting in undertreatment and avoidable suffering.^10 18^ Statements (table 2, Principle 4) reflect a shared commitment to fairness and dignity in pain care. Achieving equity requires more than policy change; it also demands the reduction of stigma and the cultivation of compassionate, inclusive practice.

#### Professional competence and continuing education in pain care

Education is presented as a moral imperative, closely tied to the duties of compassion and justice. The WHO and WMA documents consistently emphasise that (table 2, Principle 5) ethical pain care requires both clinical competence and ongoing professional education.

#### Balancing access and control in pain medicine regulation

Both organisations emphasise balancing access to essential pain medicines with appropriate regulatory safeguards. Legal frameworks must be evidence-based, aligned with human rights and sufficiently flexible to permit appropriate medical use while preventing misuse. They also advocate for national pain strategies that integrate legal regulation, clinical guidance and public input (table 2, Principle 6). They affirm a shared ethical and legal obligation: access to pain relief should not be compromised by excessive control. Ethical regulation requires both vigilance, flexibility and compassion.

#### Interdisciplinary and multimodal approaches are ethically essential in pain care

WHO and WMA documents highlight that ethical pain management requires comprehensive, multidisciplinary/interdisciplinary approaches integrating pharmacological, psychological and social interventions. These perspectives affirm that ethically sound pain care must be coordinated, multimodal and person-centred (table 2, Principle 7). Only by addressing biological, emotional, social and spiritual aspects of suffering can healthcare providers fully meet their ethical responsibilities.

#### Commitment to safety and risk awareness in pain care

Both organisations frame safety as a shared moral obligation involving patients, healthcare professionals and policy makers. They advocate for an ethical model of opioid use based on ethically guided prescribing, responsible clinical judgement and regulatory accountability (table 2, Principle 8). Risk awareness and safety are presented not as barriers to access but as integral components of compassionate and responsible pain care.

#### Respect for patient experience and autonomy

The subjective pain experience must be central to clinical decision-making, framing autonomy as a moral foundation of care. The perspectives of organisations demonstrate that respecting autonomy involves more than obtaining formal consent (table 2, Principle 9). It requires building trust, ensuring clear and compassionate communication and protecting patients’ ability to make decisions, especially in situations where they may be vulnerable.

Together, these nine principles reflect a coherent vision of value-based pain care grounded in compassion, fairness and the ethical duty to relieve suffering.

### Major barriers to equitable pain management

**Figure 2:**
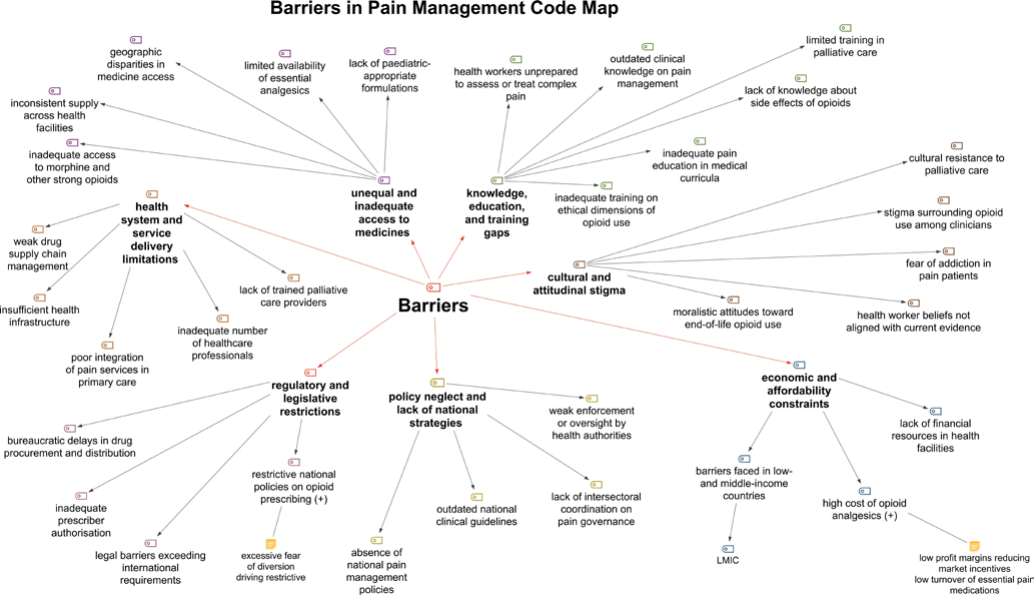
Hierarchical code map of major barriers in pain management. This figure presents a hierarchical visualisation of the major barriers identified through qualitative content analysis. The coding structure was developed using MAXQDA Analytics Pro 2022 and reflects the relationships between overarching ethical themes, subdimensions and analytical categories within the WHO and WMA documents. LMICs, low- and middle-income countries; WMA, WMA, World Medical Association.

Despite robust ethical frameworks, significant obstacles hinder equitable pain care delivery worldwide. Seven primary barrier categories emerged, reflecting structural deficiencies, restrictive regulatory environments and enduring sociocultural stigma. These findings demonstrate that ethical principles alone are insufficient without supportive systems and policies that facilitate their implementation (figure 2).

#### Unequal and inadequate access to medicines

Access to essential analgesics remains severely limited globally, disproportionately affecting LMICs.^10 17 19 20^ The WHO estimates that more than 80% of the global population lacks adequate access to pain relief and palliative care, describing this as a ‘serious public health problem’.^11 17^ Key barriers include weak procurement mechanisms, inconsistent supply across health facilities and the lack of paediatric-appropriate formulations.^2 10 16 19^

#### Regulatory and legislative restrictions

Overly restrictive national drug control laws and complex administrative procedures frequently obstruct access to effective pain relief.^14 15 20^ Licensing requirements, import limitations and prescribers’ fears of legal repercussions contribute to underprescription and undertreatment, even in cases of severe or terminal pain.^2 10 14 17^

WHO documents highlight that legal systems often exceed international drug convention requirements, resulting in additional administrative burdens and clinical uncertainty.^2 10 12 14 15^ Such barriers encompass unnecessary authorisation schemes and fragmented import and export procedures that further restrict access to essential medicines.^2 10 12 14^ Regulatory systems that prioritise preventing misuse over ensuring medical access raise serious ethical concerns and contribute to the ongoing global undertreatment of pain.^17 19 20^

#### Knowledge, education and training gaps

Persistent educational deficiencies remain a major barrier, with healthcare providers receiving inadequate instruction in pain assessment, opioid pharmacology and the ethical use of controlled substances.^3 13 15 17 20^ WHO identifies limited training in palliative care and reliance on outdated knowledge as critical obstacles.^2 4 11 13 14 16^ A lack of education regarding opioid side effects and dependence further contributes to clinical hesitation.^2 13 14 18 20^ These shortcomings are compounded by widespread ‘opiophobia’, a term describing the fear of prescribing opioids due to legal concerns, misuse or addiction.^2 3 20^

#### Cultural and attitudinal stigma

Cultural beliefs and negative attitudes towards opioid use continue hindering effective pain management.^3 10 13 20 25^ Healthcare professionals often remain reluctant to prescribe opioids despite clear clinical need, while patients may avoid reporting pain due to addiction fears or social stigma.^10 11 14 16 25^

#### Economic and affordability constraints

Economic barriers limit access, particularly in LMICs, where high out-of-pocket costs, insufficient public funding and absent subsidised medications hinder equitable access.^24 10 15^,^18 20^ Despite morphine being a low-cost essential medicine, financial constraints in procurement and distribution often lead to inconsistent availability.^10 14 17 19 20^

Additionally, hospitals in LMICs may lack the necessary financial infrastructure to support pain care services, which disproportionately affects patients in need of palliative care.^10 13 14 16 17 20^ Such financial disparities reflect broader systemic inequities and undermine the principle of justice in global pain management.^1017^,^19^

#### Health system and service delivery limitations

Structural weaknesses persist, including insufficient qualified healthcare professionals, inadequate infrastructure and weak drug supply chain management.^2 4 10 14 20^ These systemic limitations compromise the reach, quality and continuity of pain care.

#### Policy neglect and lack of national strategies

The absence of coherent national pain management strategies obstructs structural progress, with many countries maintaining outdated guidelines poorly aligned with international standards.^13 15 17 20^ WHO reports note that numerous states have failed to adopt or regularly update national pain and palliative care guidelines, thereby undermining service quality and access.^24 10 16^,^18^ Moreover, weak enforcement by health authorities and the absence of intersectoral governance frameworks contribute to fragmented oversight and poor accountability in policy implementation.^4 16 17 20^

These interconnected barriers demonstrate that equitable pain care cannot be achieved through clinical interventions alone. It requires coordinated policy reform, comprehensive professional training, increased public awareness and sustained health system investment.

### Strategic policy directions

**Figure 3:**
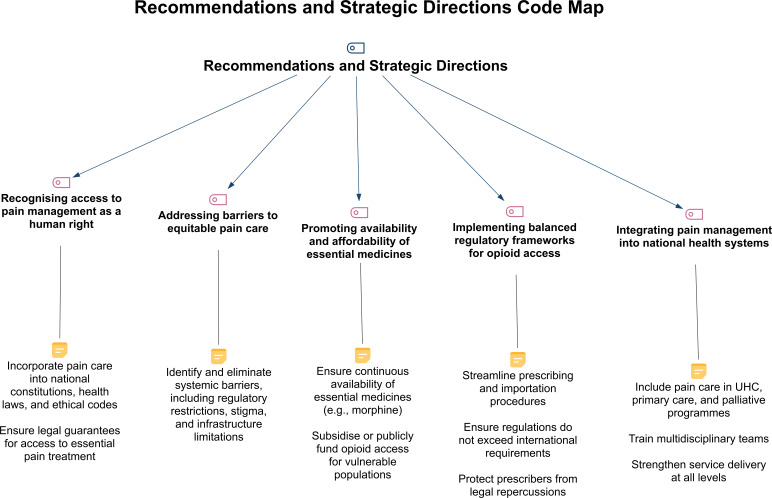
Hierarchical structure of recommendations and strategic directions in global pain policy documents. This visual map summarises the five core strategic directions identified in WHO and WMA documents and their associated implementation pathways, as revealed through qualitative content analysis using MAXQDA Analytics Pro 2022. WMA, World Medical Association.

Our analysis identifies that five strategic directions can bridge ethical principles with practical implementation (figure 3). Notably, the prominence of pharmacological interventions reflects the WHO/WMA documents’ emphasis on essential medicine access (addressing critical gaps in LMICs) but also reveals relative underemphasis on psychological and rehabilitative approaches more appropriate for chronic non-cancer pain.

1. *Recognising access to pain management as a human right:* pain treatment access should be legally protected under national laws and health policies, reframing pain management as a binding ethical and legal responsibility.^2^,^510^
2. *Addressing barriers to equitable pain care:* coordinated national and international action to eliminate access barriers, including revising restrictive regulations, strengthening health infrastructure and challenging stigma.^2^,^510^
3. *Promoting availability and affordability of essential medicines:* ensuring the reliable supply and equitable distribution of essential pain medicines requires reforming procurement and financing systems while prioritising vulnerable populations.^2^,^510^
4. *Implementing balanced regulatory frameworks for opioid access:* legal reforms should facilitate appropriate medical use while preventing misuse, for example, through simplified licensing procedures and liability protection for clinicians.^2^,^510^
5. *Integrating pain management into national health systems:* long-term improvements require embedding pain care within broader health system reforms, including universal health coverage and primary care services.^2^,^510^

These strategic directions represent a profound ethical commitment to human dignity, justice and compassion, essential for transforming pain management into universally accessible and ethically grounded care. While dignity features prominently in document language, it functions primarily as supporting justification for autonomy and non-maleficence principles rather than as an independent normative commitment requiring separate operationalisation.

These policy directions reflect the documents’ primary focus on ensuring access to essential medicines, particularly opioid analgesics, which feature prominently across WHO and WMA guidance. While this pharmacological emphasis addresses a critical global access gap, particularly morphine availability in LMICs, it represents a narrower conceptualisation of comprehensive pain care than contemporary biopsychosocial models would suggest. The relative underemphasis on psychological, social and rehabilitation interventions in these strategic directions may reflect the documents’ origins in palliative care and essential medicines advocacy. Future policy frameworks would benefit from equally robust attention to non-pharmacological pain management approaches, including psychological therapies, physical rehabilitation and community-based support structures, particularly for chronic non-cancer pain populations.^3 18 27^

### Synthesis and interpretation

This analysis reveals a coherent yet complex framework shaping global pain governance. The ethical principles form a human rights-based framework emphasising professional duty, equity and patient-centred care. However, the seven identified barriers, such as regulatory overcontrol, educational deficiencies and systemic resource constraints, create reinforcing cycles of inequity.

Notably, WHO and WMA demonstrate substantial alignment in their ethical frameworks while offering complementary perspectives: WHO emphasises public health and health systems approaches, while WMA focuses on professional ethical obligations and clinical practice standards. Both organisations converge on the need for balanced regulatory frameworks and integrated health system approaches.

The strategic directions propose a coordinated response addressing structural determinants of pain care inequity. Critically, these recommendations recognise that ethical pain care cannot rely solely on clinical interventions; it also demands multisectoral policy transformation encompassing legal frameworks, health system design and professional education.

A summary of the key ethical principles, structural barriers and strategic directions is provided in table 3.

**Table 3:** Summary of main ethical principles, major barriers and strategic policy directions

Ethical principles, barriers and strategic directions in global pain management		
Main ethical principles	Major barriers	Strategic policy directions
Access to adequate pain treatment is a fundamental human right Pain relief as an ethical duty of healthcare professionals Promoting quality of life as a central objective of ethical pain care Equitable and non-discriminatory access to pain care Professional competence and continuing education in pain care Balancing access and control in pain medicine regulation Interdisciplinary and multimodal approaches are ethically essential in pain care Commitment to safety and risk awareness in pain care Respect for patient experience and autonomy	Unequal and inadequate access to medicines Regulatory and legislative restrictions Knowledge, education and training gaps Cultural and attitudinal stigma Economic and affordability constraints Health system and service delivery limitations Policy neglect and lack of national strategies	Recognising access to pain management as a human right Addressing barriers to equitable pain care Promoting availability and affordability of essential medicines Implementing balanced regulatory frameworks for opioid access Integrating pain management into national health systems

This table offers a concise overview of the thematic structure, highlighting how ethical imperatives intersect with implementation barriers and the strategic policy directions proposed in WHO and WMA guidance.

WMA, World Medical Association.

## Discussion

Global guidelines related to pain management from the WHO and the WMA establish access to adequate pain treatment as a fundamental human right, yet significant gaps persist between these normative commitments and clinical reality. Our analysis identifies specific mechanisms through which ethical commitments translate into policy action. Beyond mere regulatory compliance, the guidelines establish a framework for proactive ethical stewardship that prioritises equity and compassion in pain care delivery. Central to this framework is the biopsychosocial model, which provides both clinical and ethical grounding by recognising pain as a multidimensional experience requiring person-centred, holistic care approaches that address biological, psychological and social dimensions simultaneously.^3 18^

Our analysis supports a model of comprehensive pain care provision that is person-centred and involves a comprehensive history, assessment, management and provision of education about pain for a patient, as well as provision of relief they find acceptable and deem safe, as defined by Harmon *et al*.^28^ Our findings regarding Principles 3 (quality of life), 7 (interdisciplinary and multimodal approaches) and 9 (respect for patient autonomy) align closely with this model, confirming that ethical pain management must address comprehensive needs, including acceptable relief, education and shared decision-making.

Our Principle 7 directly reflects this biopsychosocial orientation, with documents emphasising that ‘pain management requires a multimodal, interdisciplinary and integrated approach’ addressing ‘biological, emotional, social and spiritual aspects of suffering’.^3^ However, the strategic policy directions (figure 3) reveal an imbalance: while the ethical principles articulate comprehensive biopsychosocial commitments, the implementation strategies disproportionately emphasise pharmacological access. This disconnect suggests that translating biopsychosocial principles into policy practice remains an ongoing challenge in global pain governance.

Despite these strong ethical foundations, a persistent implementation gap undermines global pain management goals.^4 10 13 16 17 19^ This *implementation paradox* in pain care describes the situation where the populations most in need of pain care (those in resource-constrained settings) face the greatest obstacles to accessing it.^29^,^31^ As Bhadelia *et al*^32^ note, the poorest 50% of the world’s population receives only 1% of distributed opioid analgesics, while the richest 10% receives nearly 90%. Opioid consumption in many LMICs remains orders of magnitude lower than in high-income countries,^33 34^ leaving millions without adequate pain relief despite clear clinical need.^35^

Addressing this paradox requires understanding how health system design shapes implementation feasibility. The implementation of proposed strategies varies significantly across different health system types. Universal health systems may more readily integrate pain management into comprehensive coverage frameworks, while insurance-based systems face additional challenges in ensuring equitable access across diverse coverage plans. Insurance-based systems, like that of the USA, often face significant challenges in ensuring equitable access to pain management. Bohm *et al*^36^ highlight disparities in the provision, timing and type of pain treatments based on insurance status, with Medicaid patients less likely to receive restorative or integrative therapies compared with those with commercial insurance. On the other hand, universal health systems, such as in the UK, are structured to provide comprehensive coverage, enabling the integration of pain management into standard care pathways.^27^

Recent WHO policy developments validate our analysis while demonstrating continued evolution in global pain governance. The *WHO Guideline on Balanced National Controlled Medicines Policies* (May 2025) directly addresses the regulatory barriers we identified, emphasising strategies to maximise legitimate medical access while minimising misuse risks across procurement, regulation, prescribing and education. Crucially, this guidance reaffirms core ethical principles from our analysis—equitable access, professional competence and human rights-based care—indicating that these normative foundations remain central to international policy development even as implementation strategies become increasingly sophisticated.^37^

The evolution in WHO guidance reflects a shift towards more operational and context-specific implementation strategies. While maintaining ethical commitments, the 2025 guidelines provide detailed frameworks for national policy development, including specific indicators for monitoring access and safety, procurement guidelines for essential controlled medicines and training curricula for healthcare providers. This operational specificity addresses a key gap identified in our analysis: the challenge of translating broad ethical principles into actionable national policies.^37^

Despite policy advances, implementation remains constrained by deeply rooted cultural and professional barriers that resist conventional policy interventions. Cultural stigma surrounding opioid use and professional hesitancy among healthcare providers represent entrenched attitudes that persist even when supportive policies exist. These challenges highlight a critical gap in current approaches in accordance with the literature: while regulatory and structural reforms can create enabling conditions, transforming practice requires sustained, culturally sensitive interventions that address underlying beliefs about pain, suffering and opioid safety.^27 38^

Different stakeholders prioritise different aspects of the ethical framework of pain management: for instance, clinicians emphasise safety and professional competence, patients prioritise access and autonomy, while regulators focus on balanced frameworks and accountability mechanisms.^39 40^ This suggests that future policy frameworks must integrate behavioural change strategies alongside regulatory reform, employing stakeholder-specific implementation approaches that address varying priorities while maintaining coherent ethical foundations.

### Ethical tensions in global pain policy frameworks

The framework revealed in our analysis reflects inherent ethical tensions that complicate implementation. Most prominent is the justice-safety dialectic: the principle of equitable access (grounding universal pain relief as a human right) exists in ongoing tension with commitments to safety and harm prevention, particularly given the opioid crisis in high-income countries.^31^

WHO and WMA documents attempt to resolve this tension through ‘balanced’ regulatory frameworks, yet our analysis suggests this balance remains conceptually underdeveloped. Documents assert the need for equilibrium without providing clear prioritisation principles when these values conflict in practice. For instance, regulatory responses to the North American opioid crisis have prompted some high-income countries to implement restrictive prescribing protocols that, while intended to prevent addiction, inadvertently reduce access for cancer patients with legitimate pain management needs.^31^ Meanwhile, LMICs simultaneously experience severe undertreatment due to lack of essential medicine access, illustrating how safety-focused regulations in one context can paradoxically exacerbate access inequities in another, with both situations reflecting failure to balance justice and safety principles appropriately.

Similarly, the principle of ‘respect for patient experience and autonomy’ (Principle 9), while universal in articulation, raises questions about cultural adaptability. Since these guidelines are developed within Western bioethical traditions, they prioritise individual autonomy via informed consent. Therefore, this framework may not fully align with communitarian or family-centred decision-making models prevalent in many LMICs. The lack of guidance on culturally adapting these ethical requirements represents a gap in global policy. This gap suggests a need for more explicit guidance on culturally adaptive implementation that maintains core ethical commitments while respecting diverse value systems.^38 39^

The divergent stakeholder priorities identified in our analysis*—clinicians emphasising safety and competence, patients prioritising access and autonomy and regulators focusing on balanced frameworks—*reflect deeper philosophical disagreements about risk tolerance, professional discretion and the locus of decision-making authority in pain management.^39 40^ Creating unified policy frameworks that satisfy these competing priorities requires not merely technical coordination but sustained ethical dialogue about acceptable trade-offs between access, safety and autonomy.

### Critical assessment: from normative aspiration to implementation reality

Our synthesis reveals a fundamental tension in global pain governance: both WHO and WMA articulate robust, rights-based ethical frameworks, yet their own documents simultaneously acknowledge persistent, widespread implementation failure. This raises critical questions about the adequacy of current policy approaches.

First, the frameworks may suffer from insufficient attention to structural determinants. While identifying barriers (regulatory restrictions, educational gaps and resource constraints), the documents predominantly propose technical solutions (training programmes, guideline updates and regulatory streamlining) without adequately addressing the political economy of pain care inequity. The implementation paradox persists partly because essential medicines remain unaffordable, pharmaceutical markets provide insufficient incentives for low-profit analgesics and global power asymmetries concentrate resources in high-income settings.^30 32^ Ethical principles alone cannot overcome these structural realities without accompanying commitments to resource redistribution and market intervention.

Second, the frameworks reflect a technocratic orientation that may underestimate the role of cultural meaning-making in pain care. Stigma surrounding opioid use, ‘opiophobia’ among clinicians and varying cultural understandings of pain and suffering are framed primarily as educational deficits requiring correction rather than as legitimate expressions of diverse value systems requiring dialogue and negotiation. Moreover, the frameworks’ emphasis on pharmacological access, while appropriate for palliative and cancer care contexts, applies this supply-chain logic less appropriately to chronic non-cancer pain, where biopsychosocial and rehabilitative interventions should predominate. This reflects a failure to differentiate ethical imperatives across pain types.

A more critical approach would recognise that implementing ‘evidence-based’ pain management requires not merely knowledge transfer but cultural negotiation about acceptable trade-offs between pain relief, addiction risk and end-of-life care.^27 38^ Therefore, the technocratic focus of the guidelines often obscures the relational aspects of person-centred care^27 39^ framing pain management as a logistical delivery of medicines rather than a holistic response to suffering.

Third, accountability mechanisms remain notably absent from both organisations’ frameworks. Documents assert access to pain management as a ‘fundamental human right’ yet provide no mechanisms for enforcement, monitoring or redress when states fail to fulfil this obligation. Without accountability infrastructure, ethical declarations risk becoming symbolic gestures rather than enforceable commitments. The persistent gap between stated principles and implementation outcomes suggests that future policy development must move beyond normative assertion towards binding accountability frameworks with clear indicators, reporting requirements and consequences for non-compliance.

### Limitations

Several methodological considerations should inform interpretation of these findings. Our focus on WHO and WMA documents, while providing authoritative global perspectives, may not capture regional ethical frameworks or local implementation contexts.

The shared disciplinary backgrounds of both researchers, as medical doctors with ethics training, likely influenced our analytical lens in specific ways. This positioning may have led us to emphasise clinical duties and professional responsibilities, framed by a human rights perspective, alongside public health perspectives, though we attempted to balance these through explicit attention to WHO’s public health framing. Our medical training may have oriented us towards individual patient care ethics (beneficence, autonomy), yet we deliberately integrated population-level justice considerations to ensure a comprehensive interpretation of ethical principles.

Conversely, our ethics training provided conceptual tools for recognising normative frameworks that researchers from purely clinical or purely public health backgrounds might not emphasise. Broader stakeholder engagement (including patients, community advocates and policy makers) could have enriched interpretation and challenged assumptions inherent in our professional positioning. Additionally, our text-based analysis identifies policy intentions but cannot assess real-world implementation effectiveness or patient outcomes.

### Future research directions

Future research should explore national-level implementation, conduct interviews with policy makers and healthcare providers across diverse contexts and incorporate patient and community perspectives to understand how these ethical frameworks translate into lived experience.

Future research should employ mixed-methods approaches combining: (1) comparative case studies examining how specific countries with varying health system structures have translated these ethical principles into national pain policies; (2) qualitative interviews with policy makers, clinicians and patients across diverse cultural and economic contexts to understand implementation facilitators and barriers; (3) quantitative assessments correlating policy adoption with patient outcomes and access metrics and (4) participatory action research engaging communities most affected by pain care inequities to codesign culturally responsive implementation strategies. Such research would bridge the gap between normative analysis and pragmatic implementation science.

## Conclusion

Global pain management represents a critical test of international health governance: one that requires coordinated action across ethical, regulatory and clinical domains. While WHO and WMA guidelines provide robust normative frameworks grounded in human rights principles, realising equitable pain care demands systematic transformation of legal frameworks, health policies, educational curricula and clinical practices across multiple policy cycles.

The persistent implementation gaps identified in our analysis signal an urgent need for accountability mechanisms that ensure ethical commitments translate into measurable improvements in patient outcomes. Success will require sustained political commitment over multiple policy cycles, adequate resource allocation targeted towards the most underserved populations and recognition that pain relief is not merely a clinical service but a core expression of human dignity.

Achieving this transformation demands coordinated international action that addresses the implementation paradox through differentiated strategies adapted to diverse health system contexts while maintaining universal ethical standards. Only through such comprehensive approaches can the global health community bridge the gap between normative commitments and clinical reality.

This study’s unique contribution lies not merely in mapping existing frameworks but in revealing systematic gaps (particularly the absence of accountability mechanisms and insufficient attention to political–economic determinants) that explain why robust ethical principles persistently fail to translate into equitable practice. These findings point towards necessary transformations in global pain governance.

## Supplementary material

10.1136/bmjopen-2025-111913 online supplemental file 1

10.1136/bmjopen-2025-111913 online supplemental file 2

## Data Availability

All data relevant to the study are included in the article or uploaded as supplementary information.
