# Supplementary material for: How do global policy frameworks address the ethics of pain management? A qualitative content analysis of WHO and WMA documents
Source: BMJ Open. 2026 May 7;16(5):e111913. doi: 10.1136/bmjopen-2025-111913 (PMC13157773; doi:10.1136/bmjopen-2025-111913)
Supplement: online supplemental file 1 [file bmjopen-16-5-s001.docx]

**Supplementary File 1. List of WHO Documents Retrieved for Screening**

This supplementary file presents the full list of WHO documents retrieved through keyword and snowball searches conducted on 10 May 2025. A total of 109 documents were screened for relevance and inclusion in the qualitative analysis.

*Document references below follow abbreviated Vancouver formatting for brevity and clarity in supplementary listing.*

**SEARCH RESULTS**

1. Abou YZ, Alwan AAS. Guide to chemotherapy and chemoprophylaxis in bacterial infections. Alexandria: World Health Organization, Regional Office for the Eastern Mediterranean; 1993. 125 p. (WHO regional publications Eastern Mediterranean series).

2. Briggs DA. WHO guideline for non-surgical management of chronic primary low back pain in adults in primary and community care settings.

3. Henschke N, Probyn K. Protocols for systematic reviews for WHO guideline for chronic low back pain. 2022 [cited 2025 May 10]; Available from: https://osf.io/pe8bq/

4. Mock C, International Society of Surgery, editors. Guidelines for essential trauma care. Geneva: World Health Organization; 2004. 93 p. (Services).

5. World Health Assembly 67. Strengthening of palliative care as a component of integrated treatment throughout the life course: Report by the Secretariat. 2014 [cited 2025 May 10]; Available from: https://iris.who.int/handle/10665/158962

6. World Health Organization. The world health report : 2006 : working together for health. Rapport sur la situation dans le monde : 2006 : travailler ensemble pour la santé [Internet]. 2006 [cited 2025 May 10]; Available from: https://iris.who.int/handle/10665/43432

7. World Health Organization. Diagnosis and treatment. Diagnóstico y tratamiento [Internet]. 2008 [cited 2025 May 10]; Available from: https://iris.who.int/handle/10665/43827

8. World Health Organization. Guidelines for the psychosocially assisted pharmacological treatment of opioid dependence. 2009;110.

9. World Health Organization. Global action plan for the prevention and control of noncommunicable diseases 2013-2020 [Internet]. Geneva: World Health Organization; 2013 [cited 2025 May 10]. Available from: https://iris.who.int/handle/10665/94384

10. World Health Organization. Community management of opioid overdose [Internet]. Geneva: World Health Organization; 2014 [cited 2025 May 10]. 74 p. Available from: https://iris.who.int/handle/10665/137462

11. World Health Organization. Comprehensive cervical cancer control: a guide to essential practice [Internet]. 2nd ed. Geneva: World Health Organization; 2014 [cited 2025 May 10]. 364 p. Available from: https://iris.who.int/handle/10665/144785

12. World Health Organization. WHO handbook for guideline development [Internet]. 2nd ed. Geneva: World Health Organization; 2014 [cited 2025 May 10]. 167 p. Available from: https://iris.who.int/handle/10665/145714

13. World Health Organization. Planning and implementing palliative care services: a guide for programme managers [Internet]. Geneva: World Health Organization; 2016 [cited 2025 May 10]. 91 p. Available from: https://iris.who.int/handle/10665/250584

14. World Health Organization. WHO list of priority medical devices for cancer management [Internet]. Geneva: World Health Organization; 2017 [cited 2025 May 10]. 252 p. (WHO Medical device technical series). Available from: https://iris.who.int/handle/10665/255262

15. World Health Organization. Integrating palliative care and symptom relief into paediatrics: a WHO guide for health-care planners, implementers and managers [Internet]. Geneva: World Health Organization; 2018 [cited 2025 May 10]. 87 p. Available from: https://iris.who.int/handle/10665/274561

16. World Health Organization. Integrating palliative care and symptom relief into primary health care: a WHO guide for planners, implementers and managers [Internet]. Geneva: World Health Organization; 2018 [cited 2025 May 10]. 79 p. Available from: https://iris.who.int/handle/10665/274559

17. World Health Organization. Integrating palliative care and symptom relief into responses to humanitarian emergencies and crises: a WHO guide [Internet]. Geneva: World Health Organization; 2018 [cited 2025 May 10]. 107 p. Available from: https://iris.who.int/handle/10665/274565

18. World Health Organization. WHO guidelines for the pharmacological and radiotherapeutic management of cancer pain in adults and adolescents [Internet]. Geneva: World Health Organization; 2018 [cited 2025 May 10]. 138 p. Available from: https://iris.who.int/handle/10665/279700

19. WHO’s Science in 5 - Low back pain - 29 April 2024 [Internet]. 2024 [cited 2025 May 10]. Available from: https://www.youtube.com/watch?v=C1uRJXC19Bg

20. WHO. Assessing the development of palliative care worldwide: a set of actionable indicators [Internet]. 2021 [cited 2025 May 10]. Available from: https://iris.who.int/bitstream/handle/10665/345532/9789240033351-eng.pdf?sequence=1

21. WHO. WHO framework for meaningful engagement of people living with noncommunicable diseases, and mental health and neurological conditions [Internet]. 2023 [cited 2025 May 10]. Available from: https://iris.who.int/bitstream/handle/10665/367340/9789240073074-eng.pdf?sequence=1

22. WHO. Left behind in pain: extent and causes of global variations in access to morphine for medical use and actions to improve safe access Web Annex B WHO survey findings [Internet]. 2023 [cited 2025 May 10]. Available from: https://iris.who.int/bitstream/handle/10665/369238/9789240075283-eng.pdf

23. WHO. WHO guideline for non-surgical management of chronic primary low back pain in adults in primary and community care settings. Web Annex A. Contributors to the guideline. [Internet]. 2023 [cited 2025 May 10]. Available from: https://iris.who.int/bitstream/handle/10665/374379/9789240085671-eng.pdf

24. WHO. WHO guideline for non-surgical management of chronic primary low back pain in adults in primary and community care settings. Web Annex C. Evidence profile for the qualitative evidence synthesis. [Internet]. 2023 [cited 2025 May 10]. Available from: https://iris.who.int/bitstream/handle/10665/374396/9789240085695-eng.pdf

25. WHO. WHO guideline for non-surgical management of chronic primary low back pain in adults in primary and community care settings. Web Annex D. Evidence-to-decision summaries. [Internet]. 2023 [cited 2025 May 10]. Available from: https://iris.who.int/bitstream/handle/10665/374420/9789240085701-eng.pdf

26. WHO. WHO guideline for non-surgical management of chronic primary low back pain in adults in primary and community care settings. Web Annex E. List of trials by intervention. [Internet]. 2023 [cited 2025 May 10]. Available from: https://iris.who.int/bitstream/handle/10665/374422/9789240085718-eng.pdf

27. WHO. Follow-up to the Political Declaration of the High-level Meeting of the General Assembly on the Prevention and Control of Non-communicable Diseases [Internet]. 2013 [cited 2025 May 10]. Available from: https://apps.who.int/gb/ebwha/pdf_files/WHA66/A66_R10-en.pdf?ua=1

28. WHO. Cancer prevention and control in the context of an integrated approach [Internet]. 2017 [cited 2025 May 10]. Available from: https://iris.who.int/bitstream/handle/10665/275676/A70_R12-en.pdf?sequence=1&isAllowed=y

29. WHO. Cancer prevention and control in the context of an integrated approach [Internet]. 2017 [cited 2025 May 10]. Available from: https://iris.who.int/bitstream/handle/10665/275676/A70_R12-en.pdf?sequence=1&isAllowed=y

30. Amplifying the lived experience of people affected by cancer [Internet]. [cited 2025 May 10]. Available from: https://www.who.int/activities/amplifying-the-lived-experience-of-people-affected-by-cancer

31. Animation film illustrating the six components required for assessing the development of palliative care [Internet]. [cited 2025 May 5]. Available from: https://www.who.int/news/item/20-12-2021-animation-film-illustrating-the-six-components-required-for-assessing-the-development-of-palliative-care

32. Assessing the development of palliative care worldwide: a set of actionable indicators [Internet]. [cited 2025 May 10]. Available from: https://www.who.int/publications/i/item/9789240033351

33. WHO. Guidelines Development Group - Biographies of proposed members - Guidelines for the psychosocially assisted pharmacological treatment of opioid dependence and community management of opioid overdose [Internet]. 2025 [cited 2025 May 10]. Available from: https://cdn.who.int/media/docs/default-source/alcohol/bios-for-posting.pdf?sfvrsn=654d820b_5

34. WHO. BURN MANAGEMENT [Internet]. [cited 2025 May 10]. Available from: https://www.who.int/docs/default-source/integrated-health-services-%28ihs%29/csy/surgical-care/imeesc-toolkit/best-practice-safety-protocols/burn-management.pdf

35. Cancer [Internet]. [cited 2025 May 10]. Available from: https://www.who.int/news-room/fact-sheets/detail/cancer

36. Cancer EURO [Internet]. [cited 2025 May 10]. Available from: https://www.who.int/europe/health-topics/cancer/preventing-and-treating-cancer

37. Cancer Today [Internet]. [cited 2025 May 10]. Available from: https://gco.iarc.who.int/today/

38. Co-creating priorities for cancer 2030 with people affected by cancer and emergent leaders [Internet]. [cited 2025 May 10]. Available from: https://www.who.int/news-room/events/detail/2024/09/16/default-calendar/co-creating-priorities-for-cancer-2030-with-people-affected-by-cancer-and-young-leaders

39. Community management of opioid overdose [Internet]. [cited 2025 May 10]. Available from: https://www.who.int/publications/i/item/9789241548816

40. Consolidated guidelines on HIV prevention, testing, treatment, service delivery and monitoring: recommendations for a public health approach [Internet]. [cited 2025 May 10]. Available from: https://www.who.int/publications/i/item/9789240031593

41. Development of WHO Guideline on management of chronic primary low back pain in adults [Internet]. [cited 2025 May 10]. Available from: https://www.who.int/teams/maternal-newborn-child-adolescent-health-and-ageing/ageing-and-health/integrated-care-for-older-people-icope/development-of-who-guideline-on-management-of-chronic-primary-low-back-pain-in-adults

42. Endometriosis [Internet]. [cited 2025 May 10]. Available from: https://www.who.int/news-room/fact-sheets/detail/endometriosis

43. Ensuring quality cancer treatment [Internet]. [cited 2025 May 10]. Available from: https://www.who.int/activities/ensuring-quality-treatment-for-cancer

44. WHO. WHO Guideline Development Group: WHO Guideline on balanced national policies for ensuring access to and safe use of controlled medicines [Internet]. 2020 [cited 2025 May 10]. Available from: https://cdn.who.int/media/docs/default-source/controlled-substances/ensuring-balance-gdg-members-list-23012020.pdf?sfvrsn=676961e6_2

45. Feedback from Member States in response to WHO Director-General’s call to strengthen palliative care services [Internet]. [cited 2025 May 10]. Available from: https://www.who.int/news/item/09-06-2022-feedback-from-member-states-in-response-to-who-director-general-s-call-to-strengthen-palliative-care-services

46. Framework for countries to achieve an integrated continuum of long-term care [Internet]. [cited 2025 May 10]. Available from: https://www.who.int/publications/i/item/9789240038844

47. Gaza patients’ painful journey to cancer treatment [Internet]. [cited 2025 May 10]. Available from: https://www.who.int/news-room/feature-stories/detail/gaza-patients-painful-journey-to-cancer-treatment

48. Global action plan for the prevention and control of noncommunicable diseases 2013-2020 [Internet]. [cited 2025 May 10]. Available from: https://www.who.int/publications/i/item/9789241506236

49. Guidelines for essential trauma care [Internet]. [cited 2025 May 10]. Available from: https://www.who.int/publications/i/item/guidelines-for-essential-trauma-care

50. Guidelines for the psychosocially assisted pharmacological treatment of opioid dependence [Internet]. [cited 2025 May 10]. Available from: https://www.who.int/publications/i/item/9789241547543

51. Guidelines on the management of chronic pain in children [Internet]. [cited 2025 May 10]. Available from: https://www.who.int/publications/i/item/9789240017870

52. Health product and policy standards [Internet]. [cited 2025 May 10]. Available from: https://www.who.int/our-work/access-to-medicines-and-health-products/controlled-substances

53. Indicator Metadata Registry Details [Internet]. [cited 2025 May 10]. Available from: https://www.who.int/data/gho/indicator-metadata-registry/imr-details/prevalence-of-lower-back-pain-in-older-people

54. Individual Consultancy - Update of WHO Guidance document on ensuring balanced national policies for equitable and safe access to controlled medicines [Internet]. [cited 2025 May 10]. Available from: https://www.ungm.org/Public/Notice/95226

55. Individual consultancy – Qualitative synthesis of values and preferences on pharmacologic treatment of persisting pain in children [Internet]. [cited 2025 May 10]. Available from: https://www.ungm.org/Public/Notice/96357

56. Integrating palliative care and symptom relief into primary health care [Internet]. [cited 2025 May 10]. Available from: https://www.who.int/publications/i/item/integrating-palliative-care-and-symptom-relief-into-primary-health-care

57. Left behind in pain: Extent and causes of global variations in access to morphine for medical use and actions to improve safe access [Internet]. [cited 2025 May 10]. Available from: https://www.who.int/publications/i/item/9789240075269

58. Low back pain [Internet]. [cited 2025 May 10]. Available from: https://www.who.int/news-room/fact-sheets/detail/low-back-pain

59. Management of cancer [Internet]. [cited 2025 May 10]. Available from: https://www.who.int/southeastasia/activities/management-of-cancer

60. NCD stakeholder dialogue series on WHO cancer initiatives [Internet]. [cited 2025 May 10]. Available from: https://www.who.int/news-room/events/detail/2023/09/06/default-calendar/ncd-stakeholder-dialogue-series-on-who-cancer-initiatives

61. Opioid overdose [Internet]. [cited 2025 May 10]. Available from: https://www.who.int/news-room/fact-sheets/detail/opioid-overdose

62. Osteoarthritis [Internet]. [cited 2025 May 10]. Available from: https://www.who.int/news-room/fact-sheets/detail/osteoarthritis

63. Overview [Internet]. [cited 2025 May 10]. Available from: https://www.who.int/teams/mental-health-and-substance-use/alcohol-drugs-and-addictive-behaviours/overview

64. pain-guidelines-list-of-resources [Internet]. [cited 2025 May 10]. Available from: https://cdn.who.int/media/docs/default-source/documents/pain-guidelines-list-of-resources.pdf?sfvrsn=3cd7e67c_2

65. Palliative care [Internet]. [cited 2025 May 10]. Available from: https://www.who.int/europe/news-room/fact-sheets/item/palliative-care

66. Palliative care EURO [Internet]. [cited 2025 May 10]. Available from: https://www.who.int/europe/health-topics/palliative-care

67. Palliative care for children [Internet]. [cited 2025 May 10]. Available from: https://www.who.int/europe/news-room/fact-sheets/item/palliative-care-for-children

68. palliative-care-essential-facts [Internet]. [cited 2025 May 10]. Available from: https://cdn.who.int/media/docs/default-source/integrated-health-services-(ihs)/palliative-care/palliative-care-essential-facts.pdf?sfvrsn=c5fed6dc_1

69. People with medical needs are “left behind in pain” reveals new report [Internet]. [cited 2025 May 10]. Available from: https://www.who.int/news/item/16-06-2023-people-with-medical-needs-are--left-behind-in-pain--reveals-new-report

70. Planning and implementing palliative care services: a guide for programme managers [Internet]. [cited 2025 May 10]. Available from: https://www.who.int/publications/i/item/planning-and-implementing-palliative-care-services-a-guide-for-programme-managers

71. Preventing and treating cancer [Internet]. [cited 2025 May 10]. Available from: https://www.who.int/europe/activities/preventing-and-treating-cancer

72. Reducing pain at the time of vaccination: WHO position paper – September 2015 [Internet]. [cited 2025 May 10]. Available from: https://www.who.int/publications/i/item/who-wer9039

73. Safe access to morphine [Internet]. [cited 2025 May 10]. Available from: https://www.who.int/our-work/access-to-medicines-and-health-products/controlled-substances/safe-access-to-morphine

74. Scope and key question: Guideline for the management of chronic pain in children [Internet]. [cited 2025 May 10]. Available from: https://www.who.int/publications/m/item/guideline-for-the-management-of-chronic-pain-in-children

75. scope-ensuring-balance-v5-jan-2020 [Internet]. [cited 2025 May 10]. Available from: https://cdn.who.int/media/docs/default-source/controlled-substances/scope-ensuring-balance-v5-jan-2020.pdf?sfvrsn=a13d7558_2

76. SEA-NCD-108-eng [Internet]. [cited 2025 May 10]. Available from: https://iris.who.int/bitstream/handle/10665/378069/SEA-NCD-108-eng.pdf?sequence=1

77. seacangrid-brochure [Internet]. [cited 2025 May 10]. Available from: https://cdn.who.int/media/docs/default-source/searo/seacangrid-brochure.pdf

78. Statement on pain management guidance [Internet]. [cited 2025 May 10]. Available from: https://www.who.int/news/item/14-06-2019-statement-on-pain-management-guidance

79. submitted-written-statements-public-hearing-who-guideline-on-ensuring-balance [Internet]. [cited 2025 May 10]. Available from: https://cdn.who.int/media/docs/default-source/controlled-substances/submitted-written-statements-public-hearing-who-guideline-on-ensuring-balance.pdf?sfvrsn=1d3cb101_2

80. summary_who_guideline_lower-back-pain_brochure_v7 [Internet]. [cited 2025 May 10]. Available from: https://cdn.who.int/media/docs/default-source/mca-documents/ageing/lbp/summary_who_guideline_lower-back-pain_brochure_v7.pdf?sfvrsn=80aec829_1

81. The Preventable Pain Pandemic [Internet]. [cited 2025 May 10]. Available from: https://www.who.int/news-room/feature-stories/detail/the-preventable-pain-pandemic

82. Top-ranked United Kingdom palliative care services in practice – a personal story [Internet]. [cited 2025 May 10]. Available from: https://www.who.int/europe/news/item/21-06-2022-top-ranked-united-kingdom-palliative-care-services-in-practice-a-personal-story

83. Update of the WHO Guidance document: “Ensuring balance in national policies on controlled medicines, Guidance for availability and accessibility of controlled medicines” [Internet]. [cited 2025 May 5]. Available from: https://www.ungm.org/Public/Notice/96210

84. web-annex-a-survey-questionnaire [Internet]. [cited 2025 May 10]. Available from: https://cdn.who.int/media/docs/default-source/controlled-substances/web-annex-a-survey-questionnaire.pdf?sfvrsn=16114e67_3

85. webinar-slides---20230912_am [Internet]. [cited 2025 May 10]. Available from: https://cdn.who.int/media/docs/default-source/mhp-hps/webinar-slides---20230912_am.pdf?sfvrsn=22f263b4_2

86. webinar-slides---20230914_pm [Internet]. [cited 2025 May 10]. Available from: https://cdn.who.int/media/docs/default-source/mhp-hps/webinar-slides---20230914_pm.pdf?sfvrsn=d8a923e3_3

87. WER9039_505-510 [Internet]. [cited 2025 May 10]. Available from: https://iris.who.int/bitstream/handle/10665/242426/WER9039_505-510.PDF?sequence=1

88. What can be done to improve safe access to medical morphine? [Internet]. [cited 2025 May 10]. Available from: https://www.who.int/news-room/events/detail/2023/09/12/default-calendar/what-can-be-done-to-improve-safe-access-to-medical-morphine

89. WHO Expert Committee on Drug Dependence: forty-third report [Internet]. [cited 2025 May 10]. Available from: https://www.who.int/publications/i/item/9789240023024

90. WHO guideline for non-surgical management of chronic primary low back pain in adults in primary and community care settings [Internet]. [cited 2025 May 10]. Available from: https://www.who.int/publications/i/item/9789240081789

91. WHO Guidelines [Internet]. [cited 2025 May 10]. Available from: https://www.who.int/publications/who-guidelines

92. WHO Guidelines for the pharmacological and radiotherapeutic management of cancer pain in adults and adolescents [Internet]. [cited 2025 May 10]. Available from: https://www.who.int/publications/i/item/9789241550390

93. WHO issues new guidelines on the management of chronic pain in children [Internet]. [cited 2025 May 10]. Available from: https://www.who.int/news/item/01-02-2021-who-issues-new-guidelines-on-the-management-of-chronic-pain-in-children

94. WHO launches new campaign to amplify the lived experience of people affected by cancer [Internet]. [cited 2025 May 10]. Available from: https://www.who.int/news/item/18-10-2022-who-launches-new-campaign-to-amplify-the-lived-experience-of-people-affected-by-cancer

95. WHO launches new framework to support countries achieve integrated continuum of long-term care [Internet]. [cited 2025 May 10]. Available from: https://www.who.int/news/item/14-03-2022-who-launches-new-framework-to-support-countries-achieve-integrated-continuum-of-long-term-care

96. WHO Media Library [Internet]. [cited 2025 May 10]. Available from: https://who.canto.global/v/UNSGFJ2L9R/album/OFGN1?viewIndex=0

97. WHO releases guidelines on chronic low back pain [Internet]. [cited 2025 May 10]. Available from: https://www.who.int/news/item/07-12-2023-who-releases-guidelines-on-chronic-low-back-pain

98. WHO revision of pain management guidelines [Internet]. [cited 2025 May 10]. Available from: https://www.who.int/who-revision-of-pain-management-guidelines

99. WHO updates guidelines on opioid dependence treatment and overdose prevention [Internet]. [cited 2025 May 10]. Available from: https://www.who.int/news/item/09-02-2025-who-updates-guidelines-on-opioid-dependence-treatment-and-overdose-prevention

100. WHO-MVP-EMP-IAU-2019.06-eng [Internet]. [cited 2025 May 10]. Available from: https://iris.who.int/bitstream/handle/10665/325771/WHO-MVP-EMP-IAU-2019.06-eng.pdf?sequence=1

101. WHO. Strengthening of Palliative Care as a Component of Integrated Treatment throughout the Life Course. Journal of Pain & Palliative Care Pharmacotherapy. 2014 Jun;28(2):130–4.

102. BEST PRACTICES FOR THE CLINICAL MANAGEMENT OF CHRONIC PAIN. In: Guidelines on the management of chronic pain in children [Internet]. World Health Organization; 2020 [cited 2025 May 12]. Available from: https://www.ncbi.nlm.nih.gov/books/NBK566542/

103. Global Strategy to Accelerate the Elimination of Cervical Cancer As a Public Health Problem. 1st ed. Geneva: World Health Organization; 2020. 1 p.

104. CureAll Framework: WHO Global Initiative for Childhood Cancer. Increasing Access, Advancing Quality, Saving Lives. 1st ed. Geneva: World Health Organization; 2021. 1 p.

105. Framework for Countries to Achieve an Integrated Continuum of Long-Term Care. 1st ed. Geneva: World Health Organization; 2021. 1 p.

106. Guidelines on the Management of Chronic Pain in Children. 1st ed. Geneva: World Health Organization; 2021. 1 p.

107. Regional implementation framework for elimination of cervical cancer as a public health problem: 2021-2030. New Delhi: World Health Organization. Regional Office for South-East Asia; 2021.

108. WHO Expert Committee on Drug Dependence: Forty-Third Report. 1st ed. Geneva: World Health Organization; 2021. 1 p. (Technical Report Series - World Health Organization Series).

109. Left Behind in Pain: Extent and Causes of Global Variations in Access to Morphine for Medical Use and Actions to Improve Safe Access. 1st ed. Geneva: World Health Organization; 2023. 1 p.
