# Supplementary material for: How do global policy frameworks address the ethics of pain management? A qualitative content analysis of WHO and WMA documents
Source: BMJ Open. 2026 May 7;16(5):e111913. doi: 10.1136/bmjopen-2025-111913 (PMC13157773; doi:10.1136/bmjopen-2025-111913)
Supplement: online supplemental file 2 [file bmjopen-16-5-s002.docx]

Code System

| **Code System** | **Memo** | **Frequency** |
| --- | --- | --- |
| Code System |  | 4660 |
| Ethical Principles |  | 0 |
| access to adequate pain treatment is a fundamental human right |  | 8 |
| adequate pain treatment |  | 135 |
| human right | pain management is a fundamental human right. | 128 |
| inadequate pain treatment |  | 62 |
| adequate access |  | 9 |
| inadequate access | unequal and inadequate access | 6 |
| ethical duty of healthcare professionals |  | 10 |
| respecting dignity |  | 50 |
| moral imperative |  | 31 |
| ethical responsibility |  | 29 |
| compassionate care |  | 6 |
| improving quality of life |  | 47 |
| well-being |  | 85 |
| quality of life |  | 63 |
| improving health |  | 40 |
| HRQoL | health-related quality of life | 37 |
| pain reduction |  | 38 |
| improving social functioning |  | 25 |
| equitable and non-discriminatory access |  | 30 |
| non-stigmatizing |  | 85 |
| non-discriminatory access |  | 48 |
| equitable access |  | 26 |
| equity |  | 183 |
| professional competence and continuing education |  | 99 |
| training | + competency-based training | 108 |
| education |  | 80 |
| competence | + competent authorities | 67 |
| competent authority |  | 39 |
| balanced regulatory frameworks |  | 51 |
| availability of essential pain medicines | + vital pain relieving drugs | 122 |
| inappropriate use | + misuse +inappropriate prescribing +addiction +abuse | 141 |
| legal and policy systems | + legislative | 48 |
| multimodal and interdisciplinary approaches |  | 21 |
| interdisciplinary |  | 31 |
| multidisciplinary |  | 62 |
| multimodal |  | 18 |
| commitment to safety and risk awareness: |  | 34 |
| risk awareness |  | 55 |
| safe use |  | 18 |
| respect for patient experience and autonomy: |  | 32 |
| autonomy |  | 11 |
| consent | informed consent assent | 67 |
| Barriers | structural and systemic barriers | 232 |
| unequal and inadequate access to medicines |  | 8 |
| inadequate access to morphine and other strong opioids |  | 175 |
| limited availability of essential analgesics |  | 18 |
| geographic disparities in medicine access |  | 16 |
| inconsistent supply across health facilities |  | 101 |
| lack of paediatric-appropriate formulations |  | 15 |
| regulatory and legislative restrictions |  | 10 |
| restrictive national policies on opioid prescribing (+) | 4.06.2025 15:36 - ASUS Merged with code Barriers > regulatory and legislative restrictions > excessive fear of diversion driving restrictive laws | 34 |
| legal barriers exceeding international requirements |  | 15 |
| bureaucratic delays in drug procurement and distribution |  | 15 |
| inadequate prescriber authorisation frameworks |  | 13 |
| knowledge, education, and training gaps |  | 10 |
| inadequate pain education in medical curricula |  | 5 |
| lack of knowledge about side effects of opioids |  | 13 |
| outdated clinical knowledge on pain management |  | 19 |
| limited training in palliative care |  | 33 |
| health workers unprepared to assess or treat complex pain |  | 75 |
| inadequate training on ethical dimensions of opioid use |  | 7 |
| cultural and attitudinal stigma |  | 6 |
| stigma surrounding opioid use among clinicians | +opiophobia | 12 |
| cultural resistance to palliative care |  | 15 |
| fear of addiction in pain patients |  | 13 |
| moralistic attitudes toward end-of-life opioid use |  | 9 |
| health worker beliefs not aligned with current evidence |  | 11 |
| economic and affordability constraints |  | 8 |
| lack of financial resources in health facilities |  | 25 |
| high cost of opioid analgesics (+) | 4.06.2025 15:34 - ASUS Merged with code Barriers > economic and affordability constraints > low profit margins reducing market incentives  low profit margins reducing market incentives ￼ Created: ASUS, 4.06.2025 15:27 Modified: ASUS, 4.06.2025 15:28  + low turnover of essential pain medications | 26 |
| barriers faced in low- and middle-income countries |  | 176 |
| LMIC | low- and middle-income country | 62 |
| Türkiye/Turkey |  | 16 |
| health system and service delivery limitations |  | 15 |
| inadequate number of healthcare professionals |  | 61 |
| lack of trained palliative care providers |  | 17 |
| insufficient health infrastructure |  | 74 |
| poor integration of pain services in primary care |  | 133 |
| weak drug supply chain management |  | 24 |
| policy neglect and lack of national strategies |  | 9 |
| absence of national pain management policies |  | 179 |
| outdated national clinical guidelines |  | 167 |
| weak enforcement or oversight by health authorities |  | 38 |
| lack of intersectoral coordination on pain governance |  | 39 |
| Ethics |  | 32 |
| ethical |  | 96 |
| ethicist |  | 2 |
| ethical dilemma |  | 3 |
| humanity |  | 7 |
| beneficence |  | 6 |
| non-maleficence | do no harm | 3 |
| justice |  | 16 |
| double effect |  | 2 |
| palliative sedation |  | 1 |
| ASSOCIATION |  | 119 |
| NMA |  | 2 |
| IASP |  | 11 |
| Recommendations and Strategic Directions |  | 0 |
| Recognising access to pain management as a human right | Incorporate pain care into national constitutions, health laws, and ethical codes Ensure legal guarantees for access to essential pain treatment | 0 |
| Addressing barriers to equitable pain care | Identify and eliminate systemic barriers, including regulatory restrictions, stigma, and infrastructure limitations | 0 |
| Promoting availability and affordability of essential medicines | Ensure continuous availability of essential medicines (e.g., morphine)  Subsidise or publicly fund opioid access for vulnerable populations | 0 |
| Implementing balanced regulatory frameworks for opioid access | Streamline prescribing and importation procedures Ensure regulations do not exceed international requirements Protect prescribers from legal repercussions | 0 |
| Integrating pain management into national health systems | Include pain care in UHC, primary care, and palliative programmes Train multidisciplinary teams Strengthen service delivery at all levels | 0 |
| ASSEMBLY |  | 0 |
| WHA | World Health Assembly | 81 |
| WMA | World Medical Assembly | 2 |
| WMAGA | WMA General Assembly | 4 |
| UNGA | United Nations General Assembly | 26 |
| COVID-19 |  | 13 |
| Paraphrased Segments |  | 0 |
