# Supplementary material for: How do global policy frameworks address the ethics of pain management? A qualitative content analysis of WHO and WMA documents
Source: BMJ Open. 2026 May 7;16(5):e111913. doi: 10.1136/bmjopen-2025-111913 (PMC13157773; doi:10.1136/bmjopen-2025-111913)
Supplement: Supplementary file 3 [file bmjopen-2025-111913.reviewer_comments.pdf]

PEER REVIEW HISTORY

BMJ Open publishes all reviews undertaken for accepted manuscripts. Reviewers are asked to complete a checklist review form and are provided with free text boxes to elaborate on their assessment. These free text comments are reproduced below.

ARTICLE DETAILS

Title (Provisional)

How Do Global Policy Frameworks Address the Ethics of Pain Management? A Qualitative Content Analysis of WHO and WMA Documents

Authors

Coşkun, Sevim; BUKEN, Nuket Ornek

VERSION 1 - REVIEW

**Reviewer**	**1**
**Name**	**Behzadifar, Masoud**
**Affiliation**	**Iran University of Medical Sciences, Health Management and Economics Research Center**
**Date**	**26-Oct-2025**
**COI**	**None**

This manuscript presents a qualitative content analysis of WHO and WMA policy documents to elucidate the ethical frameworks and implementation barriers in global pain management. The study is timely, addresses a critical gap in global health, and is methodologically sound. The authors successfully identify a coherent set of nine ethical principles, seven major barriers, and five strategic policy directions, providing a valuable synthesis of how two major global health bodies frame this issue. The manuscript is clearly written, well-structured, and adheres to appropriate reporting standards (SRQR). While the manuscript is of high quality, the following points could be addressed to further strengthen it before publication:

Some of the nine "ethical principles" read as policy recommendations or descriptive statements of good practice (e.g., "Interdisciplinary and multimodal approaches are ethically essential," "Balancing Access and Control"). The authors should more explicitly define what constitutes an "ethical principle" in their analytical framework (e.g., drawing from Beauchamp & Childress or other frameworks) and distinguish these from derived policy imperatives. This would sharpen the conceptual clarity of the results.

The discussion could more explicitly explore the tensions and ethical dilemmas inherent in the framework. For example: Justice vs. Safety: The tension between the principle of

equitable access (justice) and the commitment to safety/risk awareness is a central ethical challenge in pain management, especially in the context of the opioid crisis. How do the documents navigate this?. The principle of "respect for patient experience and autonomy" is largely presented from a Western bioethical perspective. A brief discussion on how this principle is framed in global documents intended for diverse cultural settings would be valuable. The finding that different stakeholders prioritize different parts of the ethical framework (clinicians=safety, patients=access, regulators=balance) is excellent. This could be expanded into a brief discussion on the challenge of creating a unified policy that satisfies these divergent ethical priorities.

The limitation regarding the researchers' shared background as medical doctors with training in ethics is appropriately noted. The authors could briefly reflect on how this specific lens might have shaped the interpretation of the documents (e.g., potentially emphasizing clinical duties over public health or human rights perspectives, though the analysis seems balanced). The limitation of not assessing implementation is clear. The authors could suggest what future research (e.g., case studies of specific countries, interviews with policymakers) might look like to bridge this gap.

**Reviewer**	**2**
**Name**	**Harmon, Joanne**
**Affiliation**	**University of South Australia, Clinical and health sciences**
**Date**	**07-Nov-2025**
**COI**	**None**

An interesting article, requiring some more critical perspectives- it is descriptive and its contribution as a synthesis is not clear. Consider presentation of the findings in a more robust manner, as opposed to description- consider using PAGER for example to identify the patterns, advances, gaps, evidence and recommendations for practice.

some comments on the paper

Consider revisiting the premise of this paper as a focus on pain care provision as defined by (Harmon et al., 2019)

Pain care provision is person-centred and involves a comprehensive history, assessment, management and provision of education about pain for a patient, as well as provision of relief they find acceptable and deem safe. Consider reviewing figure 1 and being more critical in its interpretation

Fig3 has an overt narrow focus on the pharmacological use of medications for pain care provision.

Also point out the new IASP definition of pain as well.

What about consideration of the biopsychosocial aspects of pain care provision as well?

Within the documents presented is an overt biomedical model focus, need to link in within the discussion with current relevant and peer reviewed literature to move beyond a summary of documentation and accessibility to opioids. It is well known for chronic pain care provision that reliance on opioids is not considered best practice.

Needs a more robust approach such as a systematic search of the published guidelines to note where and when these documents have been used.

Page 3

Line 13

Comment is made about chronic pain- is this the focus of this review? That is management of chronic pain?

What about acute pain, cancer pain etc.

line 32

Comment is made about pain assessment, however the focus of this document is pain management, need to be clearer. This comes to light later on in page 7 whereby lines 7 and 22-31 articulates treatment of pain and assessment of pain.

Page 4

Line 30

Why the focus on palliative care? How does that relate to chronic pain. Pain care provision for end of life care differs from chronic pain, acute pain and culturally safe pain care, not withstanding pain management.

Page 18 Lines 22-36- need to integrate references into the argument, as opposed to placement at the end- this is a common theme throughout this discussion

Harmon, J., Summons, P., & Higgins, I. (2019). Nurses' culturally mediated practices influencing pain care provision for older people in acute care: Ethnographic study. Applied Nursing Research, 48, 22-29. https://doi.org/https://doi.org/10.1016/j.apnr.2019.05.010

VERSION 1 - AUTHOR RESPONSE

Reviewer 1

Some of the nine "ethical principles" read as policy recommendations or descriptive statements of good practice (e.g., “Interdisciplinary and multimodal approaches are ethically essential," "Balancing Access and Control"). The authors should more explicitly define what constitutes an "ethical principle" in their analytical framework (e.g., drawing from Beauchamp & Childress or other frameworks) and distinguish these from derived policy imperatives. This would sharpen the conceptual clarity of the results.

Answer: We appreciate this important methodological observation. We have clarified our analytical framework in the Methods section to distinguish between foundational ethical principles (e.g., beneficence, autonomy, justice) grounded in established bioethical theory, and derivative ethical obligations that specify how these principles apply to pain management policy contexts (Page 7, Lines 127-133). For example, "interdisciplinary approaches" represents a derivative obligation emerging from beneficence and non-maleficence when applied to complex pain management. We maintain that policy documents legitimately articulate principles at varying levels of abstraction, and our analysis captures this conceptual hierarchy. The nine identified principles collectively form a coherent normative framework spanning foundational commitments to implementation-oriented guidance, all of which carry ethical weight in shaping pain management policy (Page 8, Lines 150-158).

The discussion could more explicitly explore the tensions and ethical dilemmas inherent in the framework. For example: Justice vs. Safety: The tension between the principle of equitable access (justice) and the commitment to safety/risk awareness is a central ethical challenge in pain management, especially in the context of the opioid crisis. How do the documents navigate this? The principle of "respect for patient experience and autonomy" is largely presented from a Western bioethical perspective. A brief discussion on how this principle is framed in global documents intended for diverse cultural settings would be valuable. The finding that different stakeholders prioritize different parts of the ethical framework (clinicians=safety, patients=access, regulators=balance) is excellent. This could be expanded into a brief discussion on the challenge of creating a unified policy that satisfies these divergent ethical priorities.

Answer: We have substantially expanded the Discussion to address these ethical dilemmas. We now explicitly analyse the justice-safety dialectic, noting that while WHO and WMA advocate for "balanced" frameworks, the conceptual basis for resolving conflicts between equitable access and harm prevention remains underdeveloped in the documents. We have added discussion of how the autonomy principle, rooted in Western bioethical traditions, may require cultural adaptation for global implementation, highlighting a gap in current guidance. Finally, we expanded our analysis of how divergent stakeholder priorities (clinicians favouring safety, patients favouring access, regulators seeking balance) reflect deeper philosophical disagreements requiring sustained ethical dialogue beyond technical policy coordination. These additions appear in a new subsection titled "Ethical Tensions in Global Pain Policy Frameworks" in the Discussion (Page 22-23, Lines 427-458).

The limitation regarding the researchers' shared background as medical doctors with training in ethics is appropriately noted. The authors could briefly reflect on how this specific lens might have shaped the interpretation of the documents (e.g., potentially emphasizing clinical duties over public health or human rights perspectives, though the analysis seems balanced). The limitation of not assessing implementation is clear. The authors could suggest what future research (e.g., case studies of specific countries, interviews with policymakers) might look like to bridge this gap.

Answer: We have expanded the Limitations section (Page 25-26, Lines 496-513) to include explicit reflexive commentary on potential influences of our disciplinary positioning. We acknowledge that our medical backgrounds may have led us to emphasize clinical duties and individual patient ethics over population-level considerations, while our ethics training provided conceptual tools for recognizing normative frameworks. We note specific ways this positioning could have affected interpretation and emphasize that broader stakeholder engagement (including patients, policymakers, and community advocates) would have enriched our analysis. This addition enhances transparency about potential analytical biases while maintaining appropriate epistemic humility.

We also have added the “Future Research Directions” (Page 26, Lines 514-527) section to outline specific future research approaches, including comparative national case studies, stakeholder interviews across diverse contexts, quantitative policy-outcome assessments, and participatory action

research with affected communities. These suggestions address the reviewer's concern while providing concrete directions for subsequent scholarship.

Reviewer 2

An interesting article, requiring some more critical perspectives- it is descriptive and its contribution as a synthesis is not clear.

Answer: We have substantially strengthened the critical analytical dimension of the Discussion by adding a new subsection titled "Critical Assessment: From Normative Aspiration to Implementation Reality." (Page 23-25, Lines 459-495). This addition moves beyond description to critically examine three fundamental limitations in current frameworks: (1) insufficient attention to structural and political-economic determinants of inequity; (2) technocratic orientation that frames cultural differences as educational deficits rather than legitimate value diversity requiring negotiation; and (3) absence of accountability mechanisms to enforce stated human rights commitments. This critical synthesis clarifies our contribution: we not only map existing ethical frameworks but identify systematic gaps that explain persistent implementation failure, pointing toward necessary transformations in global pain governance.

This study systematically analyses ethical principles across WHO and WMA pain management documents using rigorous QCA methodology, revealing not only the normative frameworks but also, critically, the conceptual tensions and accountability gaps that explain why robust ethical commitments fail to translate into equitable practice globally

Consider presentation of the findings in a more robust manner, as opposed to description- consider using PAGER for example to identify the patterns, advances, gaps, evidence and recommendations for practice.

Answer: We appreciate this methodological suggestion. The PAGER framework offers valuable structure for systematic reviews of empirical literature. Our qualitative content analysis of policy documents employs a complementary analytical logic: thematic identification of normative patterns (ethical principles), systematic barrier analysis (gaps), extensive textual evidence (Table 2), and policy-oriented synthesis (strategic directions). While we have not relabeled sections using PAGER terminology, our analytical approach addresses similar dimensions (patterns, advances, gaps, evidence, and recommendations) in a manner appropriate for normative policy document analysis. Future systematic reviews examining pain management implementation literature would benefit from explicit PAGER application.

Consider revisiting the premise of this paper as a focus on pain care provision as defined by (Harmon et al., 2019).

Answer: We appreciate the reviewer directing us to this person-centred conceptualization. Following this suggestion, we have integrated Harmon et al.'s person-centred care framework explicitly in the Discussion (page 17, paragraph 1), showing how our Principles 3, 7, and 9 align with their emphasis on acceptable relief, education, and shared decision-making. We have clarified in the Discussion that our ethical principles provide normative foundations that should inform person-centred pain care models such as that articulated by Harmon et al. (Page 19-20, Lines 362-368).

Pain care provision is person-centred and involves a comprehensive history, assessment, management and provision of education about pain for a patient, as well as provision of relief they find acceptable and deem safe. Consider reviewing figure 1 and being more critical in its interpretation.

Answer: We have added critical analytical commentary following Figure 1's introduction in the Results section (Page 8, Lines 159-168). This addition examines what the hierarchical structure

reveals about the frameworks' strengths (evolution toward rights-based approaches) and limitations (unresolved tensions between competing principles, potential overemphasis on professional duties relative to patient agency). This critical lens enhances the interpretive depth of our visual representation.

Fig3 has an overt narrow focus on the pharmacological use of medications for pain care provision.

Answer: We acknowledge this important observation. We have added explicit commentary in the Results section noting that the strategic directions' emphasis on medicine access, while addressing a critical global gap (particularly morphine availability in LMICs), represents a narrower conceptualization than contemporary biopsychosocial approaches would suggest (Page 16, Lines 292-296; Lines 318-328). We note that this pharmacological focus likely reflects the documents' origins in palliative care and essential medicines advocacy, and we explicitly call for future policy frameworks to provide equally robust attention to psychological, rehabilitative, and community-based interventions—particularly for chronic non-cancer pain. This addition appears following the presentation of the five strategic directions and acknowledges a significant limitation in current global pain governance frameworks.

Also point out the new IASP definition of pain as well.

Answer: We have incorporated reference to the IASP's revised 2020 definition of pain in the Introduction (Page 3, Lines 61-67), which explicitly acknowledges pain's biopsychosocial dimensions (biological, psychological, and social factors). This addition strengthens our conceptual foundation and aligns with our subsequent discussion of biopsychosocial approaches in pain management policy. The citation has been added to the reference list.

What about consideration of the biopsychosocial aspects of pain care provision as well?

Answer: Our analysis identifies biopsychosocial dimensions as central to the ethical frameworks articulated by both organizations. Specifically, Principle 7 (interdisciplinary and multimodal approaches are ethically essential in pain care) explicitly addresses the need for comprehensive approaches integrating biological, psychological, and social interventions. We discuss the biopsychosocial model extensively in the Discussion section, noting its role as both clinical and ethical foundation for person-centred care. However, as we now acknowledge following the reviewer's observation about Figure 3, there exists a concerning disconnect: while ethical principles articulate biopsychosocial commitments, strategic policy directions disproportionately emphasize pharmacological access. We have made this tension explicit, recognizing that translating biopsychosocial principles into implementation practice remains an ongoing challenge (Page 20, Lines 369-376).

Within the documents presented is an overt biomedical model focus, need to link in within the discussion with current relevant and peer reviewed literature to move beyond a summary of documentation and accessibility to opioids. It is well known for chronic pain care provision that reliance on opioids is not considered best practice.

Answer: We have substantially strengthened integration of contemporary literature throughout the Discussion section (Pages 18-25), engaging with: implementation science literature documenting the "implementation paradox," empirical studies on global opioid access disparities, health systems research on insurance-based vs. universal coverage models, recent WHO policy developments (ref 37), medical education literature on training gaps, and person-centred care frameworks. This integration situates our document analysis within broader scholarly discourse and demonstrates how our findings align with, contradict, or extend current understanding of global pain care challenges.

Needs a more robust approach such as a systematic search of the published guidelines to note where and when these documents have been used.

Answer: We appreciate this suggestion, which points toward an important complementary research approach. However, such systematic citation tracking and implementation assessment would constitute a fundamentally different study design, closer to systematic review methodology with implementation science focus, rather than the qualitative content analysis of policy documents we have undertaken. Our study's purpose is to analyse the normative ethical frameworks articulated within WHO and WMA documents themselves, not to trace their subsequent uptake or impact. We have clarified in our Limitations section that our analysis "identifies policy intentions but cannot assess real-world implementation effectiveness" and we explicitly call for future research including "national-level implementation studies" and "interviews with policymakers and healthcare providers" to examine how these frameworks translate into practice. We believe document analysis and implementation tracking are complementary but methodologically distinct approaches, both valuable for understanding global pain governance.

Page 3, Line 13: Comment is made about chronic pain - is this the focus of this review? That is management of chronic pain? What about acute pain, cancer pain etc.

Answer: Our analysis encompasses pain management policies across diverse clinical contexts, including acute, chronic, cancer-related, and palliative pain. While we mentioned chronic pain in the Introduction as one significant global burden, the included WHO and WMA documents address pain management broadly, with particularly robust coverage of cancer pain and palliative care contexts. We have revised the Introduction to clarify that our scope includes multiple pain types and note which categories feature most prominently in available international guidance (Page 3, Lines 51-60).

Page 3, Line 32: Comment is made about pain assessment, however the focus of this document is pain management, need to be clearer. This comes to light later on in page 7 whereby lines 7 and 22-31 articulates treatment of pain and assessment of pain.

Answer: We have clarified that pain assessment is discussed as a component skill necessary for effective pain management, not as a separate focus. Healthcare professionals require competency in both assessment and treatment; assessment is the foundation upon which management decisions rest. Our revised text now states "professional training in pain assessment and management" (Page 3, Lines 68-72) to clarify this integrated relationship.

Page 4, Line 30: Why the focus on palliative care? How does that relate to chronic pain. Pain care provision for end of life care differs from chronic pain, acute pain and culturally safe pain care, not withstanding pain management.

Answer: We have added clarification in the Methods section (Page 5, Lines 99-107) explaining that WHO's pain management guidance has historically developed primarily within palliative and cancer care contexts, where pain relief is a core therapeutic objective. While palliative care traditionally addresses life-limiting illness rather than chronic non-cancer pain, the ethical principles (rights-based access, equity, professional duty) and implementation barriers (regulatory restrictions, education gaps, stigma) identified in these documents apply broadly across pain management contexts. We acknowledge that chronic non-cancer pain may raise distinct ethical considerations requiring additional policy development, but palliative care-focused documents provide valuable foundational insights into pain management ethics more generally.

Page 18, Lines 22-36: need to integrate references into the argument, as opposed to placement at the end- this is common theme throughout this discussion.

Answer: We have revised citation practices where appropriate, particularly in the Discussion (Pages 19-20), to integrate references more naturally within the argumentative flow (e.g., Bhadelia et al., Harmon et al.), while maintaining standard Vancouver clustering for lists of supporting documents.
